# Carotid artery plaques and unilateral spatial neglect in the elderly

**DOI:** 10.1097/MD.0000000000018998

**Published:** 2020-01-24

**Authors:** Yixiao Wu, Yue Dai, Yanli Jia, Shuhang Yu, Siyuan Xu, Wei Wang

**Affiliations:** a Department of Ultrasound B, Beishan Community Health Service Center; b Department of Clinical Psychology and Psychiatry/ School of Public Health, Zhejiang University College of Medicine, Hangzhou, China; c Department of Psychology, Norwegian University of Science and Technology, Trondheim, Norway.

**Keywords:** carotid artery plaque, elderly population, fall injury, unilateral spatial neglect

## Abstract

The causes for falls in the elderly are varied, and visual spatial neglect could be 1 contributing factor. Further, the presence of a carotid artery plaque, especially on the right side, might influence the visual spatial attention of the elderly.

Our aim was to identify the intrinsic association between carotid plaques and lateralization of spatial attention in the elderly. Further, we sought to understand and potentially prevent the consequences of unilateral spatial neglect such as injury from falls.

Participants aged 64 to 93 years were divided into a group with carotid artery plaque(s) of the right side or both sides (BOTH, n = 38; and 9/ 38 were right side only) and a group without right-side carotid artery plaque(s) (LEFT, n = 53). Participants were asked to perform a line bisection task and undergo doppler ultrasonography examinations.

Contrary to expectations, compared to LEFT, the mean index and net scores of the line bisection errors in BOTH were significantly less leftward, but the mean diameter of the right-side common carotid artery in BOTH was significantly larger.

Our results indicate that the presence of carotid plaque(s) might be linked to increased risk of falls in the elderly. The attenuated spatial neglect in participants with right-side carotid artery plaque(s) might be due to compensatory carotid artery dilatation.

## 1 Introduction

The risk of fall is increased in the elderly population,^[1]^ and fall itself is a leading cause of disability and hospitalization in these patients.^[2]^ Factors causing fall in the elderly are numerous, including functional disabilities, chronic disease conditions,^[3]^ and unilateral spatial neglect. Individuals with deficits of visual spatial attention have problems reporting, responding, or locating to new or meaningful stimuli,^[4]^ for example, not being able to see the food on 1 side while eating.

Factors contributing to unilateral spatial neglect include stroke, brain tumors, and other cerebral lesions.^[5]^ Unilateral chronic stroke, for instance, is caused by insufficient blood supply to the diseased hemisphere, leading to an enhanced leftward visual attention in patients with right side cerebral stroke; that is, their right space is neglected.^[6]^ Anatomically, the internal carotid artery supplies blood to the forebrain,^[7]^ and the occipital lobe (via the direct continuation of the middle cerebral artery). On the other hand, a carotid plaque is a risk factor for acute ischemic stroke, acute brain infarction, transient ischemic attack, and myocardial infarction,^[8]^ and the length and vessel diameter of the carotid plaque are thought to be predictors of blood flow or velocity.^[9]^ Thus, the carotid plaque, through influencing blood flow, might affect the visuospatial attention.

Considering that the right hemisphere is more dominant in the spatial attention than the left hemisphere, and that spatial attention processing or orientation predominantly activates the right fronto-parietal areas,^[10]^ we speculated that increased spatial neglect in the elderly was intrinsically linked to the occurrence of carotid plaque, especially the right-side. A very simple technique, the line bisection performance, helps to detect visual spatial neglect.^[11–13]^ Patients with left-side neglect typically bisect horizontal lines to the right-side of the veridical center.^[5,14–16]^

Therefore in the present study, we hypothesized:

(1) Those elderly participants with right-side carotid plaque would bisect lines more leftward, and
(2) The frequency or magnitude of their bisection errors would be associated with carotid artery diameters and blood flow. Elderly participants were also asked to undergo a Doppler ultrasonography examination of the morphology of the carotid artery, plaque, and blood flow velocity.

## 2 Methods

### 2.1 Participants

A total of 91 participants with one or multiple plaques in their carotid cavities, were recruited from a community hospital of Hangzhou, China, when receiving their annual health checkups. Participants were grouped into a group without right-side carotid artery plaque(s) (LEFT) (n = 53; 13 men and 40 women; mean age: 72.8 years ± 7.1 standard deviation; age range: 57 - 84 years) if they had no plaque in the right-side carotid artery, or grouped into a group with carotid artery plaque(s) of the right side or both sides (BOTH) (n = 38; 25 men and 13 women; mean age: 77.8 ± 6.1; age range: 64 - 93) if they had 1 or more plaques in the right-side carotid artery or in both right- and left-side carotid arteries. There was no difference in gender (*χ*^2^ = 1.02, *P* = .31), but there was a difference in age (*t* = 3.48, *P* = .001) between these 2 groups. Moreover, participants were confirmed to have no organic brain lesions according to the recent magnetic resonance imaging or computed tomography scans. They were also confirmed to have no neurological or psychiatric problems such as substance use disorder or an eating disorder, and had not ingested alcohol, drugs, or medication for at least 72 hours prior to the test, as determined via a semi-structured clinical interview. The study was approved by a local ethics committee (ZGL201409-1-3), and all participants gave their written informed consent to participate in the study.

Using a Chinese translation of the Edinburgh Handedness Inventory,^[17]^ we determined the handedness of each participant. Each inventory item was rated with either 1, 2 or 3 according to the left-hand, either left or right, or right-preference. All elderly participants scored from 29 to 36, thus were considered to be moderate or strong right-handers.

### 2.2 Carotid morphology, hemodynamics and serum concentration

A plaque was defined as a local protuberance extending more than 0.5 mm from the arterial lumen or exceeding 50% (or more than 1.5 mm) of the surrounding normal thickness of the intima-media. If there were multiple plaques in the right carotid artery, we measured the largest 1 in that individual participant. Meanwhile, we measured the diameters of common carotid arteries and the internal carotid arteries on both sides, and the related hemodynamic parameters such as blood pressure, end-systolic velocity of the common carotid artery, end-diastolic velocity of the common carotid artery, end-systolic velocity of the internal carotid artery, end-diastolic velocity of the internal carotid artery, resident index of the common carotid artery, and the resident index of the internal carotid artery. In addition, we measured the serum concentrations of triglycerides, total cholesterol, high density lipoprotein cholesterol, and low density lipoprotein cholesterol.

### 2.3 Line bisection performance

There were 8 lines which were drawn in black and oriented horizontally, with a length of 95 to 146 mm, randomly placed on an A4 size paper, 1 line under another line, and their distances from the paper margins were different, so their centers were not aligned. The paper was always centered at the midsagittal plane of the elderly participants. Without measuring or folding the paper, the elderly participants were instructed to use their right hand to make a mark indicating the center of the line in order to divide these eight lines into 2 halves. No restrictions on the movement of the head or eyes, and no time limit were imposed on participants.

The distance of the line bisecting mark was measured from the veridical center to the nearest millimeter. The frequency of directional errors, irrespective of the magnitude, was measured using the index of line bisection error (Index). This was calculated as (Right - Left)/(Right + Left); positive values indicate errors to the right and negative values indicate errors to the left. The magnitude of line bisection deviation was calculated as the algebraic sum of the distance of marks from the veridical center. This statistic is called the net of line bisection errors (Net). Thus, positive values of Net indicate errors to the right and negative values indicate errors to the left.

### 2.4 Statistical analyses

In 2 groups of participants, the Student *t* test was applied to the mean hemodynamic, carotid artery morphology, and serum parameters, and the Chi-square test was used to compare gender distributions. The analysis of covariance (ANCOVA) (adjusted for age) and post-hoc Student *t* test were applied to the mean Index and Net. The relationship between Index/ Net and parameters of serum, artery morphology and hemodynamics, was assessed by the partial correlation test (adjusted for age). A *P* value less than .05 was considered to be significant.

## 3 Results

The ANCOVA detected no statistically significant difference (*t* = 0.06 - 0.34) in blood pressure, serum triglyceride, or cholesterol concentrations between the 2 groups of participants. Additionally, there was no difference (*t* = 0.11 - 0.95) in blood flow velocities between the 2 groups (Table 1).

**Table 1:**
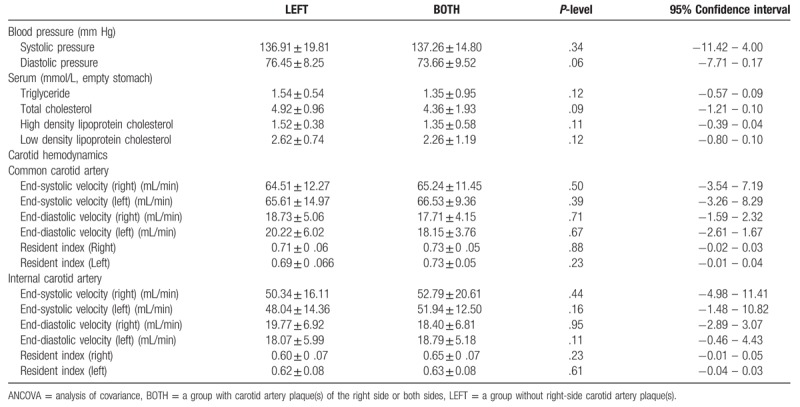
Differences on factors contributing to the cerebral blood flow in the elderly participants with no carotid artery plaque(s) (LEFT, n = 53) and with right side only or both sides (BOTH, n = 38) carotid artery plaque(s), analyzed by ANCOVA (adjusted for age).

In contrast, the ANCOVA detected a statistically significant difference in the mean diameters of the right-side common carotid artery (F [1,88] = 4.769, mean square error [MSE] = 0.006, *p* = .032), the Index (F [1, 86] = 10.188, MSE = 2.838, *P* = .002) and the Net (F [1, 86] = 9.217, MSE = 26.074, *P* = .003) in the 2 groups of participants (Table 2). The BOTH group (0.89 ± 0.08) had a larger-diameter right-side common carotid artery than LEFT group (0.84 ± 0.07) did. The BOTH group (-0.10 ± 0.53) scored less frequently leftward than the LEFT group (-0.41 ± 0.53). The BOTH group (-0.37 ± 2.08) in addition, bisected less severely leftward than the LEFT group (-1.16 ± 1.49) (Table 2).

**Table 2:**
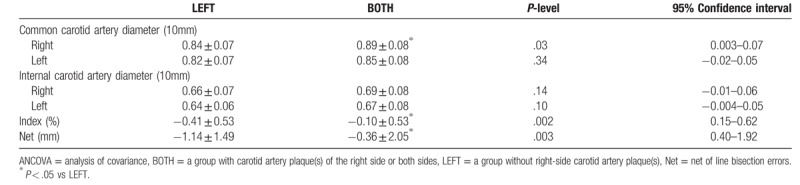
Differences on diameters of carotid artery and the line bisection errors in the elderly participants with no carotid artery plaque(s) (LEFT, n = 53) and with right side only or both sides (BOTH, n = 38) carotid artery plaque(s), analyzed by ANCOVA (adjusted for age).

The Partial Correlation test did not detect any significant correlation between line bisection errors (Index or Net) and serum, hemodynamic or artery morphological parameters in the LEFT group (rs = -.32 - .22, *ps* > .05), or in the BOTH group (rs = -.31 - .28, *ps* > .05).

## 4 Discussion

All participants bisected leftward, and the LEFT group bisected significantly more leftward, signifying that they had right spatial neglect. The BOTH group placed their marks less leftward, and their right carotid artery diameters were significantly larger than those of the LEFT group. The spatial attention of those with plaques on the right/both sides was more laterally biased than that of the left-side plaque(s) group, which rejects our first hypothesis. Additionally, the bisection errors were not correlated with either the vessel diameter nor the blood flow velocity, which also rejects our second hypothesis. However, the participants in the BOTH group of our study had a larger vessel diameter of the carotid artery, which might compensate for the possible blood flow deficiency related to the plaque formation. We anticipate that this might contribute to the unexpected findings herein, reducing the impact on spatial attention function.

Plaques mostly grow at the bifurcation of the common carotid artery,^[18]^ and protrude to the lumen, and can lead to arteriostenosis to the point of occlusion.^[19]^ Thus, artery plaques ruin intima structure in the artery. For instance, 86% patients with internal carotid artery stenosis with stenotic restriction greater than 70%, and have cerebral hypoperfusion.^[20]^ In addition, the degree of stenosis of the internal carotid artery greatly influences its blood flow.^[9]^ In brief sumary, the existence of artery plaques has a significant impact on blood flow, often resulting in cerebral hypoperfusion.

Long-term insufficient blood supply causes pathological damage to the brain, and, subsequently, to spatial attention. At the same time, the insufficient blood supply causes a compensatory response, which promotes the physiological expansion of the common carotid artery, so that the blood flow increases to meet the supply of the brain. Similar results were previously reported, where the expansive remodeling ratio was significantly related to the carotid artery stenosis induced by plaque.^[21]^ These relationships support our speculation that the presence of plaque is likely to promote carotid artery dilatation. If the blood supply is insufficient for a long period of time, the common carotid artery continues to irreversibly expand, developing a pathological state. Moreover, this is potentially why the right-side carotid artery has been diluted in our BOTH group. However, there is no plausible explanation for why the left-side plaque executed no similar influence on the dilution of the left-side carotid artery in the LEFT group.

Researchers have shown that left-side spatial neglect is a disabling neurological condition which typically results from right hemisphere damage.^[22]^ The internal carotid artery is divided into anterior and middle cerebral arteries after entering brain, which supply blood to 60% of the frontal brain. Hence, the blood flow of the internal carotid artery greatly influences the cerebral perfusion greatly. For instance, in right hemisphere-lesioned patients, neglect was most frequently associated with lesions involving (in descending order) the temporal, parietal, frontal, occipital lobes, basal ganglia, and thalamus.^[23]^ Therefore, in the present study, we might speculate that when the internal carotid artery blood flow is deficient, the visuospatial neglect might occur, as indicated by the error scores of Index and Net in the 2 groups.

However, there were several design flaws in our present study. First, due to the cross-sectional nature of this study, we cannot determine the causes of the carotid plaque and its longitudinal effects on spatial attention. Second, we only calculated the hemodynamics of the artery, other factors such as the plaque volume might exert an influential function on the spatial neglect. Third, our participants were all elderly, and younger healthy volunteers would be a useful comparison to the current results. Fourth, we did not measure the prevalence of falling in our participants, which might offer a meaningful causative-explanation, especially in a future longitudinal design. However, our study provides data which may be useful for increasing our understanding of spatial neglect and risk of the fall injury in the elderly. For instance, the plaques in the left side of carotid artery might also increase the incidence of falling in the senile population, and a carotid Doppler ultrasonography examination might be beneficial as a routine screening test to identify patients at high risk.

## Acknowledgments

The authors thank all participants who had contributed much of their time and energy to the present study.

## Author contributions

**Conceptualization:** Wei Wang.

**Data curation:** Yixiao WU, Yue DAI, Yanli JIA, Shuhang YU, Siyuan XU.

**Formal analysis:** Yixiao WU, Yue DAI.

**Methodology:** Wei Wang.

**Project administration:** Yixiao WU, Yue DAI.

**Supervision:** Wei Wang.

**Writing – original draft:** Yixiao WU, Yue DAI.

**Writing – review & editing:** Yixiao WU, Yue DAI.

Wei Wang orcid: 0000-0002-6822-8162.
